# Phase I dose-escalation trial of irinotecan with continuous infusion 5-FU first line, in metastatic colorectal cancer

**DOI:** 10.1038/sj.bjc.6602173

**Published:** 2004-09-28

**Authors:** M P Saunders, M Hogg, B Carrington, A-M Sjursen, J Allen, J Beech, R Swindell, J W Valle

**Affiliations:** 1 Christie Hospital, Wilmslow Road, Manchester, M20 4BX, UK

**Keywords:** CPT-11, 5-FU, irinotecan, Lokich, metastatic colorectal cancer

## Abstract

This single-centre phase I trial was designed to determine the maximum tolerated dose of irinotecan and the recommended dose to use in combination with a fixed dose of 5-fluorouracil (5-FU) administered as a protracted venous infusion, for the first-line treatment of metastatic colorectal cancer (CRC). Tolerability and efficacy were secondary end points. In all, 22 patients, median age 57 years, were treated with escalating, weekly doses of irinotecan (50, 75, 100 and 85 mg m^−2^) in combination with 250 mg m^−2^ 5-FU administered as a continuous infusion. All patients had measurable disease. The combination was well tolerated up to an irinotecan dose of 75 mg m^−2^. However, three out of five patients at the 100 mg m^−2^ irinotecan dose level had their dose reduced due to multiple grade 2 toxicities, and eventually one patient stopped treatment due to grade 3 diarrhoea and multiple grade 2 toxicities. Subsequent patients were recruited at an irinotecan dose level of 85 mg m^−2^. The overall response rate was 55%, comprising one complete and 11 partial responses (PRs). Six patients also achieved sustained stable disease (SD), giving a clinical benefit (complete response/PR/SD) response of 82%. The median duration of response was 238 days (8.5 months) and median time to progression was 224 days (8.0 months). Two patients who achieved PRs underwent partial hepatectomies. Thus, irinotecan (85 mg m^−2^) combined with a continuous infusion of 5-FU (250 mg m^−2^) is an active and well-tolerated regimen for the treatment of metastatic CRC. It represents an effective treatment for patients who require close supervision and support, throughout their initial exposure to chemotherapy for this disease, and this dose combination was recommended for an ongoing phase II study.

Colorectal cancer (CRC) is second in the league of cancer deaths in developed countries. Approximately 25% of patients have liver metastases at presentation and eventually 50% of newly diagnosed patients will succumb to metastatic disease. The liver is the most common site of disease spread, and approximately 60% of patients with liver metastases have no evidence of extrahepatic disease (Faivre *et al*, 1995). However, only 10–20% of these patients are candidates for surgical resection (Adam, 2003), and although there are new treatment techniques evolving, such as radiofrequency ablation (Nordlinger and Rougier, 2002) and the regional delivery of chemotherapy by hepatic arterial infusion (Kemeny and Ron, 1999; Kemeny, 2000), systemic chemotherapy still plays the major role in the treatment of metastatic CRC.

The fluoropyrimidine 5-fluorouracil (5-FU) has been the cornerstone of therapy for CRC for over 30 years and during this time has been the subject of extensive investigation with regard to the optimisation of its use. In combination with the biochemical modulator leucovorin (folinic acid (FA)), it remains the reference drug for the treatment of CRC in adjuvant, first-line and second-line settings, with response rates of 22 and 14% for continuous infusion and bolus 5-FU regimens, respectively (Meta-analysis group in cancer, 1998). Modulation with FA prolongs the effect of 5-FU, conferring benefit similar to that seen with a continuous infusion. Two of the most common combinations of 5-FU and FA have been directly compared (de Gramont *et al*, 1997). The European infusional 5-FU/FA approach (de Gramont regimen) was deemed to be preferable to the North American bolus 5-FU/FA strategy developed at the Mayo Clinic (de Gramont *et al*, 1997). The response rate for the de Gramont regimen was significantly higher, 32.6 *vs* 14.4%, than for the Mayo regimen (*P*=0.0004). Progression-free survival (PFS) was also increased, although the median overall survival was similar (Mayo, 56.8 weeks; de Gramont, 62 weeks, *P*=0.067). The de Gramont regimen was also better tolerated with significantly less mucositis. However, the future of 5-FU-based therapy appears to lie in its combination with newer agents with nonoverlapping mechanisms of action, and several agents have demonstrated good efficacy in the treatment of patients with metastatic CRC. These agents include the topoisomerase-I inhibitor irinotecan (CPT-11) (Wiseman and Markham, 1996) and the platinum compound oxaliplatin (Wiseman *et al*, 1999). In phase II/III studies, irinotecan has demonstrated consistent antitumour activity when used as second-line therapy in 5-FU-resistant metastatic disease, with response rates in the range 11–23% (Wiseman and Markham, 1996). Irinotecan has also demonstrated a significant survival advantage with better quality of life in two phase III studies comparing irinotecan with best supportive care (Cunningham *et al*, 1998) and with infusional 5-FU/FA (Rougier *et al*, 1998) following progression on 5-FU-based therapy. The first-line, single-agent activity of irinotecan (Conti *et al*, 1996; Pitot *et al*, 1997; Rougier *et al*, 1997), coupled with its novel mechanism of action, led to its development in combination with 5-FU-based regimens for the first-line therapy of metastatic CRC. Two pivotal phase III trials, one in Europe (Douillard *et al*, 2000) and the other in the US (Saltz *et al*, 2000), investigated the efficacy of irinotecan in combination with one of two infusional 5-FU/FA regimens (AIO or de Gramont) (Douillard *et al*, 2000) or a bolus 5-FU/FA regimen (Saltz *et al*, 2000). The efficacy results for the two trials were remarkably similar with significant improvements in response rates, time to progression (PFS), and overall suvival for the irinotecan/5-FU/FA combination arms of both trials when compared with the corresponding 5-FU/FA control arms. The most significant finding from both trials was that irinotecan, in combination with 5-FU/FA, irrespective of the 5-FU/FA regimen used, conferred a significant survival advantage. As a result, irinotecan in combination with 5-FU/FA was approved for the first-line therapy of advanced and metastatic CRC in both Europe and the US. More recently, continuous infusional 5-FU alone (the Lokich regimen) has been shown to be equivalent to the 5-FU/FA de Gramont regimen in terms of overall survival (Maughan *et al*, 2002). The Lokich regimen was generally well tolerated, although there was a greater incidence of hand–foot syndrome (HFS) compared with the de Gramont regimen. However, patient quality of life (QOL) was similar with both regimens. Importantly, the Lokich regimen could be administered for half of the cost of the de Gramont regimen (Hale *et al*, 2002). Thus, the present study aimed to identify the optimal dose of irinotecan for use in combination with the Lokich regimen of infusional 5-FU alone (i.e. without FA) for the treatment of patients with metastatic CRC. It was also postulated that the tolerability would be improved by a small but regular dose of irinotecan. Response rates, to ensure that efficacy was maintained, and toxicity data were also recorded.

## MATERIALS AND METHODS

### Patient selection

Patients were eligible for inclusion in this single-centre, phase I, open-label, dose-escalation trial if they had histologically confirmed, advanced adenocarcinoma of the colon or rectum, with inoperable, measurable, metastatic disease (synchronous or recurrent). Selection criteria included: age ⩾18 years, Karnofsky performance status (PS) ⩾70 and a life expectancy of 3 months. Patients were required to have adequate haematological function (haemoglobin ⩾10 g l^−1^, neutrophils ⩾1.5 × 10^9^ l^−1^, platelet count ⩾150 × 10^9^ l^−1^), acceptable hepatobiliary function (serum bilirubin <1.25 × upper limit of normal (ULN), alkaline phosphatase <5 × ULN, aspartate aminotransferase <3 × ULN) and adequate renal function (estimated Cockcroft clearance >50 ml min^−1^).

Patients were excluded if they had received prior chemotherapy for metastatic disease or adjuvant chemotherapy within the 6 months prior to study entry, had a concurrent uncontrolled medical illness or previous or current malignant disease likely to interfere with protocol treatments and comparisons. Patients could not have partial or complete bowel obstruction, chronic diarrhoea or inflammatory bowel disease, any confirmed abnormality of biliary transport, previous transplantation surgery or a recent history of uncontrolled angina or cardiac arrhythmias. The trial was conducted with full local ethical committee approval according to the accepted standards of good clinical practice and in agreement with the latest version of the Declaration of Helsinki. All patients provided written informed consent. Pretreatment baseline evaluations included a complete medical history and physical examination, complete blood cell count and blood chemistry plus complete tumour assessment both radiologically and by a tumour marker (carcinoembryonic antigen (CEA)).

### Treatment and dose escalation

Treatment was commenced as soon as possible after registration and within 4 weeks of the assessments for eligibility and disease evaluation. Initially, 5-FU was administered as a continuous, protracted infusion, at a dose of 200 mg m^−2^ day^−1^, for 2 weeks. If grade 0 or 1 stomatitis or HFS were the only toxicities to occur, then the 5-FU dose was increased to 250 mg m^−2^day^−1^ for the remainder of the trial unless further toxicity occurred that required a dose reduction.

The starting dose of irinotecan was 50 mg m^−2^ administered as a 30-min infusion once weekly. The planned dose levels were 50, 75, 100 and 125 mg m^−2^, with increases in the weekly dose to be made in a step-wise fashion in cohorts of three patients. Intrapatient dose escalation was not allowed. All patients were assessed weekly for both haematological and nonhaematological toxicity according to the National Cancer Institute-Common Toxicity Criteria (NCI-CTC v 2). Patients could only be entered at the next dose level if no dose-limiting NCI-CTC grade 3 or 4 toxicity was observed. If one of the three patients developed grade 3 or 4 nonhaematological toxicity (e.g. diarrhoea, vomiting, infection) within eight cycles from the start of the study, a further three patients were enrolled at that dose level. Similarly, if one patient developed grade 4 haematological toxicity, a further three patients were enrolled at that dose level. In the absence of further incidences of grade 3/4 toxicity in the expanded cohort, patients could be enrolled at the next dose level. If all three patients at a particular dose level of irinotecan developed significant haematological or nonhaematological toxicity, treatment was stopped and the dose level below was taken as the MTD. If patients developed grade 3 or 4 stomatitis or HFS, then the 5-FU dose was reduced by 50 mg m^−2^.

Chemotherapy was administered for eight cycles prior to re-assessment of measurable metastatic disease. One cycle was defined as 1 week of chemotherapy with irinotecan on day 1 and continuous infusion of 5-FU. In patients who responded or who developed stable disease (SD), the treatment was continued for a further eight cycles, and the response assessment then repeated. If a patient continued to have SD or a response, then treatment could be continued for a further eight cycles, that is, up to a maximum of 24 cycles of chemotherapy. Treatment was discontinued in the case of disease progression, unacceptable toxicity or withdrawal of patient consent. Concomitant medication included prophylactic antiemetics (dexamethasone 8 mg and ondansetron 8 mg both intravenously (i.v.)), with the irinotecan infusion followed by metoclopramide 10–20 mg orally every 4 h as required. Some patients also required 24–48 h of ondansetron (8 mg bid) and dexamethasone (4–8 mg bid) if nausea was a problem. Delayed diarrhoea was treated early and aggressively with loperamide, with the addition of oral ciprofloxacin if it persisted for more than 24 h.

### Toxicity assessment and evaluation of response

Patients were seen weekly, when their symptom control was assessed and they were subjected to a physical examination. A complete blood count and biochemistry profile was also performed. Patients were assessed every 3 weeks using the tumour marker CEA, and the first, last and lowest CEA levels during the study were recorded.

Tumour response was assessed radiologically every eight cycles. All radiological assessments were conducted using the same method employed for the primary assessment. Most patients had CT scans; however, in some patients disease was followed by chest X-ray (CXR) and/or ultrasound if the tumours could be clearly visualised using this method (CT: 14 patients, ultrasound liver: six patients, CXR: two patients). Assessment without CT scans was allowed in the earlier parts of the trial since the main aim of this phase I study was to determine the dose/schedule to take forward to the phase II part of the study. Apart from one subject, all patients who received 85 or 100 mg m^−2^ were assessed using CT scans. All of the radiological investigations were reviewed at the end of the study by a single consultant radiologist without the benefit of the original reports. Any major discrepancies were re-checked and these measurements were used in this study. All responses were reported according to World Health Organisation criteria. The date of relapse was taken to be the date of radiological confirmation of relapse. For responders, the duration of response was taken from the time of the start of treatment to the time of first documented disease progression. For all patients, time to progression was the time from the start of treatment to the time of documented disease progression.

## RESULTS

Between 8 May 2001 and 23 July 2002 (14 months), 22 patients (15 males and seven females), with confirmed measurable, metastatic CRC, were included into this single-centre, phase I dose-escalation trial. The demographic and baseline disease characteristics of the patients at study entry are summarised in Table 1

**Table 1:** Patient characteristics

No. of patients	22
Median age, years (range)	57 (34–70)
Sex (M/F)	15/7
	
*Primary tumour site*	
Colon	5
Rectum	11
Rectosigmoid	6
	
*Karnofsky performance status*	
100	2
90	16
80	3
70	1
<70	0
	
*No. of sites of metastatic disease*	
1	10
>1	12
	
*Sites of metastatic disease*	
Liver	7
Lung	2
Liver and lung	8
LN	1
Liver and LN	2
Lung and LN	2

LN=lymph node.

. Seven patients had metastasis to the liver only, two to the lung only and one to lymph nodes only. All the remaining patients had multiple sites of disease involvement. Overall, 17 out of 22 patients had measurable liver disease, 12 out of 22 measurable lung disease and five out of 22 measurable lymph node disease. All patients were evaluable for safety, toxicity and response.

In total, 397 cycles of irinotecan therapy were administered to 22 patients. All patients received both irinotecan and 5-FU at each dose level. Seven patients completed 24 weeks of therapy. Six patients stopped treatment due to progressive disease (PD), four due to toxicity (one of whom went on to have a liver resection), four were planned and one patient underwent a liver resection.

### Maximum tolerated dose

Three patients received a total of 39 cycles of irinotecan therapy at the 50 mg m^−2^ dose level, and six patients received 110 cycles of irinotecan therapy at the 75 mg m^−2^ dose level for irinotecan. There were no dose reductions at either dose level, but five and 13 cycles were delayed at the 50 and 75 mg m^−2^ dose levels, respectively, due to toxicity or patient choice (i.e. holidays, Table 2

**Table 2:** Irinotecan dose escalation

		**Irinotecan cycles (*n*)**		**Dose reduction (*n*)**		**Dose delay (*n*)**			
**Dose of irinotecan (mg m^−2^)**	**No. of patients**	**Total**	**Median (range)**	**Patients**	**Cycles**	**Patients**	**Cycles**	**Median dose intensity up to eight cycles (%)**	**Median dose intensity up to 16 cycles and >8 cycles (%)**
50	3	39	13 (10–15)	0	0	2	5	100	74.9
75	6	110	18 (7–24)	0	0	4	13	79.4	76.7
100	5	106	21 (16–24)	3	46	5	17	68.7	66.0
85	8	142	18 (4–24)	0	0	5	17	88.9	84.2
Total/overall	22	397		3	46	16	52	87.5	76.3

). One patient developed grade 3 nausea and vomiting at the 75 mg m^−2^ dose level, necessitating a week's delay and the need for improved antiemetics. At the 100 mg m^−2^ irinotecan dose level, 106 cycles of irinotecan therapy were administered to five patients (Table 2). At this dose level, all five patients had dose delays during 17 cycles, and three out of five patients had dose reductions during 46 cycles. One patient stopped treatment due to grade 3 diarrhoea and multiple grade 2 toxicities. The other four patients all had multiple grade 2 toxicities, in particular, lethargy and diarrhoea (Table 3

**Table 3:** Worst toxicity per patient (all cycles) by irinotecan dose level considered related to irinotecan in combination with continuous infusion 5-FU

	**Dose of irinotecan (mg m^−2^)**											
	**50**			**75**			**100**			**85**		
Number of patients	3			6			5			8		
Number of cycles	39			110			106			142		
												
NCI-CTC toxicity grade	2	3	4	2	3	4	2	3	4	2	3	4
												
*Toxicity*												
Diarrhoea	3			3			8	1		3		
Weight loss	1			2			1			2		
Neutropenia	0			4			0			3		
Leucopenia	0			5			0			8		
Lethargy	1			0			8			1		
Anaemia	1			13			1			5		
Stomatitis	1			0			1			0		
Cardiac toxicity	0			0			0			0	1^a^	
ALP	5			3			0			0		
Bilirubin	1			2			1			0		
HFS^b^	0			0			0			2		
Nausea^c^	0			0	1		0			1		
Thrombosis	1			0			0		1	0		

NCI-CTC=National Cancer Institute-Common Toxicity Criteria; ALP=alkaline phosphatase; HFS=hand-foot syndrome.

a Related to 5-FU infusion.

b Six patients had grade 1 HFS.

c 11 patients had grade 1 nausea.

), and it became clear that this dose level was not well tolerated. In view of the unacceptable level of toxicity observed at the 100 mg m^−2^ dose level of irinotecan, a new ethical committee approval for a lower dose was applied for, and all subsequent patients received irinotecan at a dose of 85 mg m^−2^. The use of multiple grade 2 toxicities in the determination of the MTD was considered to be a practical approach and one that provided a realistic measure of tolerability. The occurrence of several grade 2 toxicities can have a profound impact on the ability and willingness of patients to continue with therapy. This dose was chosen because it represented only a small increase over the apparently acceptable 75 mg m^−2^ dose level. At the 85 mg m^−2^ irinotecan dose level, 142 cycles of irinotecan therapy were administered to eight patients.

### Toxicity assessments

The combination of irinotecan and continuous infusion 5-FU was well tolerated up to an irinotecan dose level of 100 mg m^−2^ (Table 3). No patient at the 50 mg m^−2^ dose level experienced any grade 3 toxicities. One patient stopped treatment after nine cycles of therapy because of progressive disease and one patient stopped therapy at cycle 16 to undergo a successful right hemihepatectomy. This patient eventually developed pulmonary metastases. One patient developed grade 3 nausea and vomiting at the 75 mg m^−2^ dose level, necessitating a week's delay after cycle 9 and the need for improved antiemetics. Two patients stopped treatment early because of progressive disease and the remaining patients stopped treatment after 24 cycles. At the 100 mg m^−2^ dose level, two out of the five patients treated experienced grade 3/4 toxicities (grade 3 diarrhoea in one patient and grade 4 thrombosis in one patient). The patient with grade 3 diarrhoea stopped treatment at cycle 16 and subsequently went on to undergo a liver resection. This patient is well and is considered to have achieved a complete response (CR) 8 months after stopping chemotherapy. At the revised irinotecan dose level of 85 mg m^−2^, one of the eight patients treated experienced grade 3 cardiac toxicity, considered to be related to 5-FU, and the treatment was stopped at week 4. One patient had grade 3 weight loss, while another two patients treated at this dose level experienced grade 2 5-FU toxicity: one patient had the 5-FU dose reduced after 3 weeks to 200 mg m^−2^ and the other patient did not have the 5-FU dose stepped up to 250 mg m^−2^. Overall, irinotecan-related grade 3/4 toxicity was seen in four patients. One patient at 75 mg m^−2^ developed grade 3 nausea and vomiting, while one patient at 100 mg m^−2^ developed grade 3 diarrhoea. The other two grade 3/4 toxicities (cardiac and thrombosis) were not felt to be related to the escalating doses of irinotecan. With the exception of 5-FU-induced cardiac toxicity (cycle 4), all grade 3/4 toxicities occurred after cycle 9.

### Response

Out of the 22 patients, 12 achieved a response (one CR and 11 partial response (PR)) to yield an overall response rate of 55%. Responses according to irinotecan dose level are shown in Table 4

**Table 4:** Antitumour efficacy

	**Starting dose of irinotecan (mg m^−2^)**			
**Response**	**50 (*n*=3)**	**75 (*n*=6)**	**100 (*n*=5)**	**85 (*n*=8)**
Complete response	—	—	1	—
Partial response	2	2	3	4
Stable disease	—	2	1	3
Progressive disease	1	2	—	1
				
Objective response rate	55%			
Clinical benefit (CR/PR/SD) (22 evaluable patients)	82%			
				
				
Median duration of response (*n*=12)	238 days			
Median time to progression (*n*=22)	224 days			

. Six patients had SD and the clinical benefit to this cohort was 82% (CR+PR+SD). The overall median duration of response was 238 days (8.5 months). There was an interesting correlation between radiological response and CEA change. Altogether, 92% of patients (11 out of 12) who responded to this treatment developed a greater than 50% reduction in CEA value. On the other hand, only one of the four patients whose disease progressed achieved at least a 50% reduction in CEA. The situation for patients with SD was less clear. These data therefore suggest that CEA provides a guide to outcome in those patients who respond well or progress on treatment. However, no firm conclusions can be made from this cohort of only 22 patients. The median time to progression was 224 days.

## DISCUSSION

Irinotecan is routinely administered intravenously either as a single agent (Cunningham *et al*, 1998; Rougier *et al*, 1998; Rothenberg *et al*, 1999; Van Cutsem *et al*, 1999) or in combination with bolus or infusional 5-FU/FA regimens, using a variety of different schedules (Douillard *et al*, 2000; Saltz *et al*, 2000), for the treatment of advanced CRC. The objective response rate of 55% and time to progression of 8 months obtained in the present study compare favourably with the response rates of between 39 and 49% and times to progression of approximately 7 months reported for irinotecan in combination with 5-FU/FA in the two pivotal phase III trials. Also, a clinical benefit response was observed in 82% of patients. The dose-limiting toxicities (DLTs) following irinotecan therapy, irrespective of schedule, are diarrhoea or diarrhoea and neutropenia (Vanhoefer *et al*, 2001). Severe grade 3/4 diarrhoea and neutropenia are estimated to occur in approximately 20% of patients. The DLTs in the present study were diarrhoea and lethargy, consistent with those expected for this combination (Douillard *et al*, 2000; Saltz *et al*, 2000). There were no treatment-related deaths and there was a very low level of neutropenia (seven instances at grade 2 in 228 cycles; none at grade 3/4). Eight patients (36%) experienced grade 1 (six patients) or grade 2 (two patients) HFS attributable to treatment with continuous infusion of 5-FU. The 85 mg m^−2^ dose level was well tolerated with considerably less diarrhoea and lethargy than the 100 mg m^−2^ dose level (Table 3). Only one episode of grade 3 toxicity related to the chemotherapy was found in the 100 mg m^−2^ cohort (five patients), and under normal circumstances this would have been deemed to be acceptable and a dose escalation would be considered. However, it became obvious that patients were not coping with this level of treatment and were experiencing many grade 2 toxicities. At this point, concerns were being raised in North America about the Saltz regimen (Rothenberg *et al*, 2001; Sargent *et al*, 2001) and it was therefore decided to reduce the irinotecan dose in this study. We feel this was the correct decision and this study emphasises the need to look at toxicity as a whole rather than simply the number of patients with grade 3 or 4 toxicities. It is noteworthy that grade 3/4 toxicities tended to present late in the study (cycles 9–19), suggesting a cumulative toxic effect. The data from the MRC CRO6B study showed that patients had more toxic effects and serious adverse events if treatment was given continuously rather than intermittently (Maughan *et al*, 2003). Our findings are therefore in agreement with this study and are consistent with the belief that patients with advanced CRC fair less well the longer they receive continuous chemotherapy. It is also important to consider that, in the case of some phase I studies, escalation to the next dose level is sometimes made after only one cycle of treatment. This may be a little too early to be certain that the toxicity attributed to the previous dose level is acceptable. When this study was initially envisaged, it was felt that a small weekly dose of irinotecan would be well tolerated and efficacious. This was certainly the case and the excess cost of the irinotecan was partially reduced by the omission of FA and the reduction in the number of infusion pumps required to administer this treatment. The major disadvantage of this treatment is the need for weekly visits to hospital to receive this therapy. However, this can be an advantage for patients who are quite symptomatic, either from the disease or the treatment itself. These appointments allow time for frequent review of symptom control and support. The quality of life issues raised here have been evaluated in more detail in the follow-on phase II study, which is near to completion.

This study commenced before the widespread availability of oral fluoropyrimidines such as capecitabine. The advent of such drugs is a major step forward in terms of patient acceptability and convenience. However, the only available randomised data that prove their efficacy are from trials where the oral agent has been compared to *bolus* 5-FU regimens rather than infusional ones (Hoff *et al*, 2001; Van Cutsem *et al*, 2001). The European infusional 5-FU/FA approach, pioneered by de Gramont *et al* (1997), was found to be better than the North American bolus 5-FU/FA strategy developed at the Mayo Clinic. The response rate for the de Gramont regimen was significantly higher at 32.6% compared to 14.4% with the Mayo Clinic regimen (*P*=0.0004). Progression-free survival was also increased; however, the median overall survival was similar (Mayo Clinic, 56.8 weeks; de Gramont, 62 weeks, *P*=0.067). The de Gramont regimen was also much better tolerated with significantly less mucositis. Weekly low-dose irinotecan has been shown to be well tolerated in the EORTC 40986 study where irinotecan (80 mg m^−2^) was combined with the AIO infusional 5-FU regimen (Köhne *et al*, 2003). This phase III randomised trial of 430 patients, which was presented at the American Society of Clinical Oncology (ASCO) meeting in 2003, gave a PFS of 8.8 months and an overall survival of 20.1 months. The DLT was diarrhoea, necessitating a reduction in the dose of 5-FU. At the lower dose, grade 3/4 diarrhoea occurred in 23% of patients on the combined arm, compared to 16% of patients who received just AIO. Weekly irinotecan (75 mg^−2^) has also been combined with continuous capecitabine (1000 mg m^−2^ bid^−1^) (Burris *et al*, 2004). As with the previous study, the main toxicity was diarrhoea, which occurred in 17% of patients (grade 3/4). Both of these studies emphasise that low-dose weekly irinotecan is relatively well tolerated and the Kohne study also shows that it is very effective. Our study, using a similar dose of irinotecan (our recommended dose: 85 mg m^−2^), supports these findings. Nonetheless, it is likely that the convenience and acceptability of oral fluoropyrimidines will prevail and they will become more widely used in combination with irinotecan and oxaliplatin at the expense of the infusional 5-FU regimens. However, until the phase III results of these combinations are published, showing good efficacy and tolerability (and the funding for such regimens is made available), weekly irinotecan and a continuous infusion of 5-FU remains a well-tolerated and effective therapy.

In summary, the preliminary data from this study are very encouraging both in terms of efficacy and tolerability. This is an effective treatment for patients who require close supervision and support, throughout their initial exposure to chemotherapy for advanced CRC.
